# Effects of alloy composition in traditional Japanese *shakudo* patination

**DOI:** 10.1371/journal.pone.0289728

**Published:** 2023-11-17

**Authors:** Agnese Benzonelli, Ian C. Freestone, Marcos Martinón-Torres

**Affiliations:** 1 McDonald Institute for Archaeological Research, University of Cambridge, Cambridge, United Kingdom; 2 UCL Institute of Archaeology, London, United Kingdom; 3 Department of Archaeology, University of Cambridge, Cambridge, United Kingdom; Universiti Malaysia Pahang, MALAYSIA

## Abstract

Japanese craftspeople have dominated the art of patinating copper-alloys since the 15^th^ century, using precise alloy compositions and complicated patination processes in different hot solutions to create a variety of colours on swords fittings such as *tsuba*. While this complex tradition is increasingly popular in the East, the reasons behind the choices made by craftspeople in the selection of the components of the alloys and are still not fully understood. This paper investigates the effect of different alloying elements (tin, gold, and silver) on the resulting patina. Experimental results are compared with optical and compositional analyses on historical Japanese artefacts, confirming the effects of the different alloying elements on the patina characteristics and colour. The absence of tin and the presence of gold limit the growth of an oxide layer and promote the formation of a thin patina characterised by a smooth appearance without visible grains. Therefore, a limited thickness of the patinas is a key aspect for the production of the desired colour and appearance of the patinas. The first colorimetric analysis on historical Japanese artefacts demonstrates the influence of gold, silver and tin in the final patina colour, validating the observations in the experimental replicas.

## 1 – Introduction

No other culture has been able to create and play with coloured metal alloys like that of Japan. Beginning at the end of the 15^th^ century, Japanese craftspeople created an entire class of alloys called *irogane*, or ‘coloured alloys’. These were made by adding small amounts of other metals to copper, and then simmering the resulting alloy in a chemical solution containing copper salts, alum, acids and other ingredients [e.g. 1–5]. Combining a variety of alloy compositions and solution ingredients, Japanese craftspeople were able to create coloured patinas ranging from a pale brown to a deep black [3–8]. By inlaying *irogane*, as well as gold, silver, and lacquers, Japanese artisans created artefacts with complex and visually impressive designs and patterns. *Irogane* were mainly used for sword fittings, and especially for decorating the *tsuba*, the hand guard of Japanese swords [e.g. 9, 10].

*Shakudō* is thought to have been the main *irogane* alloy [e.g. 8, 11]. It comprised a series of copper-gold alloys containing gold concentrations ranging between 0.25 and 10 wt.% which, once patinated, presented a bluish-black hue. Its dark colour made it the perfect base for inlaying with other *irogane*, but it was also used to inlay other non-metal materials, to create striking contrasts. While the scientific literature describes *shakudo* as an alloy made only with copper and gold, Japanese historical sources also report the presence of a low percentage of silver.

*Shibuichi* is another category of patinated copper-based alloys, but with about 25 wt.% silver (the word ‘shibuichi’ means ‘a quarter’ in Japanese). *Shibuichi* is also known as ‘foggy or hazy silver’, a suitable description for the colour of the patina, which ranges from brown to bright silver [3, 4, 11, 12]. Other alloys belonging to the *irogane* class are *shinchū* (Cu + 15–20wt.% Zn + 2–5wt.% Pb) and *sentoku* (Cu + 8wt.% Sn + 13wt.% Zn + 6wt.% Pb), which after patination become yellowish-brown [4]; as well as *karakane* (Cu + 2–8wt.% Sn + 5–15wt.% Pb) and *kuromido* (Cu + <3wt.% As), which develop a dark brown to black coloured patina and for this reason often replaced *shakudō*, except in the case of high quality items [4, 9, 11, 12].

Since the discovery of *irogane* in the West in the 19^th^ century, *shakudō* and *shibuichi* are the two alloys that have attracted the most attention from archaeologists and scientists, who have tried to explain the ’sacrificial use’ of gold and silver as alloying agents in the complex *irogane* patination process.

A similar patinated alloy containing precious metals has been used from early modern Chinese artefacts such as vases, boxes, and inkwells and called *wu-tong* [13, 14]. This material differed from *irogane* due to the incorporation of both gold and silver in the same copper alloy, and it was patinated with the use of either a chemical solution or the perspiration of the hands [14].

Experimental and analytical studies of *shakudo* have been very limited. They have shown that the patinated layer is very thin (2–3 um), and rich in cuprite (cuprous oxide, Cu_2_O) [4, 5, 15]. It has also been hypothesised that the dark colour of the patina layer is due to gold nanoparticles embedded in the alloy surface, which absorb light [4, 5, 15]. However, the mechanism controlling the patina formation and hence the colour is not fully understood.

The colour of the *shakudo* was first described by Pumpelly as “bluish-black” in 1866 [16, p.44], and then reported by numerous authors afterwards. This blue shade of the *shakudo* distinguish this material from other darker patinas (for example, the greyish-black patinas in high-tin bronzes) and it is one of the most peculiar, famous, interesting, and mysterious aspects of these patinas.

This paper focuses on the patination technique used to produce *shakudō* and *shibuichi* and builds on preliminary experiments discussed in Benzonelli *et al*. [17]. We report experimental replication of patinas in order to investigate the relationship between production technology, alloy composition (in particular, the presence of gold, silver, and tin), and the physico-chemical characteristics of the patina. Subsequently, the experimental patinas are compared to historical *shakudō* and *shibuichi* to determine why Japanese craftspeople made certain technological choices in the selection of alloy compositions.

## 2 – Method

### 2.1: Experimental patination

The experimental method was based on the results of the pilot study described in Benzonelli *et al*. [17]. We produced replica tokens of eight alloys, four of which were included in the original experiments: Cu_100_, Cu_95_Sn_5_, Cu_97_Au_3_, and Cu_89_Sn_5_Au_3_Ag_3_ plus four new compositions: Cu_97_Ag_3_, Cu_92_Sn_5_Au_3_, Cu_92_Sn_5_Ag_3_, and Cu_94_Au_3_Ag_3_, where subscripts indicate the composition in wt.%. There have been some suggestions that the presence of arsenic may also modify patina colour in *shakudo* (e.g. [18]), but because of health and safety considerations, it could not be included in this study.

In that previous work, it was demonstrated that the superficial work (abrading or polishing) carried out on a copper alloy object prior to patination should achieve an optimum between too smooth a surface, which does not allow formation and adhesion of the patina, and too rough a surface, which results in an uneven patina. As a consequence, for the present experiment, a medium degree of surface abrasion (ground to 600 grit with silicon carbide paper) was used.

The earlier experiments [17] also showed that the most successful patinas, comprising compact and homogeneous layers of fine grains covering the entirety of the sample surface, were obtained using a copper acetate treatment solution, the main documented ingredient to have been used in Japanese patination. Therefore, in the present study, the patination was performed on a hotplate with a copper acetate solution (0.4 g in 100 ml of deionised water). The solution temperature was maintained at 80°C for 90 minutes for the eight alloys selected. After patination, the tokens were gently washed with deionised water.

### 2.2: Museum artefacts investigation

**Fig 1:**
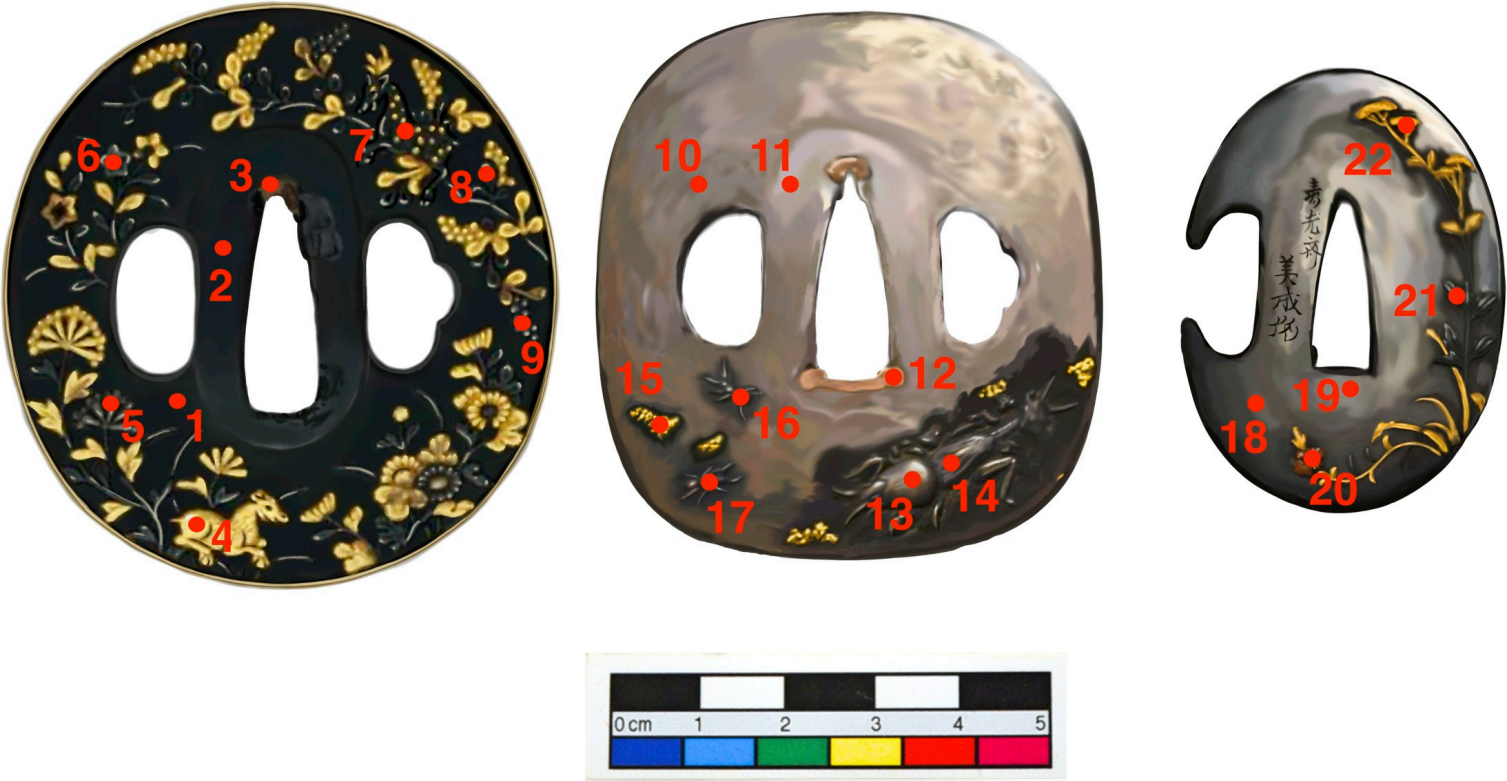
Drawing of the Japanese artefacts from the British Museum investigated in this study, with the analytical spots, reported in Table 2. The link to the online Catalogue for the three objects are the following: https://www.britishmuseum.org/collection/object/A_TS-244, https://www.britishmuseum.org/collection/object/A_1952-0211-31, https://www.britishmuseum.org/collection/object/A_1958-0730-56-d.

Following the experiments, historic Japanese *tsuba* were analysed to ground-truth the observations made on the experimental tokens. Three *tsuba* from the British Museum collections with *shakudō* base or inlay were selected (Fig 1). These are: BM-TS244 (*shakudō* base), BM1952,0211.31 (*shakudō* ants), and BM1958,0730.56.d (*shakudō* leaf). Artefact details are listed in S1 Table in S1 File.

### 2.3: Analytical methods

Experimental patinas were analysed with the dual aims of characterising their differences, and guiding the development of an analytical protocol in a manner which satisfies museum constraints for minimally invasive analysis. Analytical techniques included colorimetry, optical microscopy, X-ray fluorescence (XRF), scanning electron microscopy with energy dispersive X-ray spectroscopy (SEM-EDS), X-ray diffraction (XRD), Raman spectroscopy, and ultraviolet-visible (UV-vis) spectroscopy.

Museum artefacts were analysed with colorimetry, optical microscopy, and portable XRF (pXRF). Five XRF and three colorimeter measurements were taken for each artefact.

The analytical method used on both experimental and historical patinas was as follows.

#### 2.3.1: Colorimetry

Was used to measure the colour of the patina and, indirectly (by comparing results on several spots), its uniformity and coverage on the sample. The instrument used was a X-Rite i1 Pro portable reflection spectrodensitometer using a 45°/0° geometry with no filter and a 4.5mm measurement aperture, resulting in a 6.0 mm measurement diameter. The machine was calibrated with the white standard ceramic chip provided by the manufacturer. Measurements were performed with the illuminant D65/10 for 5 seconds in the *specular component included* (SCI) mode. The colours were recorded in the L*a*b* system, where L defines the lightness, with (from 0 –black- to 100 –white); a* represent colours from green (-ve) to red (+ve) and b* blue (-ve)to yellow (+ve), along with the reflectance spectra for 380-730nm, at 10nm intervals. Experimental samples were measured four times in different areas.

#### 2.3.2: Optical microscopy (OM)

Was used on he flat surface of the unprepared sample to evaluate the colour and the appearance of the patina at high magnification. A Leica DM-LM optical microscope with 10, 20, 50, and 80X magnification was used.

#### 2.3.3: X-ray fluorescence spectrometry (XRF)

Was used to analyse the elemental compositions of experimental alloys before and after patination. Analysis was performed using a portable Olympus Innov-X Delta in Alloy mode with a single beam for a live time of 40 seconds with 40kV accelerating voltage, and with quantification based on fundamental parameters. A collimator was used to produce a 5 mm spot on the sample. This provided consistency between the analyses on the patinas and the analyses performed on museum samples, where patinated decorations are often small and require a collimated beam. Certified reference materials (bronze alloys) were used as secondary standards. The analyses of the standards are included in S2 Table in S1 File. Analytical totals were normalised to 100 wt.%.

#### 2.3.4: Scanning electron microscopy with energy dispersive X-ray spectroscopy (SEM-EDS)

Was used to investigate the microstructure and the composition of the sample on the surface and in cross section. A Hitachi S-3400N was used with an accelerating voltage of 20 kV and beam current of 10 nA. X-ray spectra were collected using an Oxford Instruments EDS with INCA software, with an acquisition time of 120 s and a detector dead time of 30–40%. Certified reference materials (bronze alloys) were used as secondary standards to check the accuracy of the analyses (included in S3 Table in S1 File) while a cobalt standard was used to calibrate the electronics and correct for instrumental drift. Analytical totals were normalised to 100 wt.%. Analyses were carried out on both the flat surface of the unprepared sample and in polished cross section. For the latter, small specimens were cut from the samples, mounted in epoxy-resin blocks and polished down to a 0.25 µm finish.

#### 2.3.5: X-ray diffraction (XRD)

Analysis was performed to identify the phases in the patinas using a Rigaku MiniFlex diffractometer with Bragg-Brentano geometry and with the following instrumental settings: Cu anode (Kα1 = 1.5406), current of 15 mA, and 40 kV voltage. The analysis was performed from 5.0° to 90.0° 2θ, with a step size of 0.05° and at a rate of 10 seconds/step. Spectra treatment and qualitative analysis were performed using DIFFRAC.EVA software [19] and the PDF2-ICDD database [20].

#### 2.3.6: Raman spectroscopy

Is superficial (approximately 2 μm) and is therefore particularly suitable for thin layers such as patinas. A Renishaw Raman Microscope equipped with violet laser (442 nm) and notch filter was used for analysis. The analytical conditions were varied according to the material response and were: objective (100X-500X), accumulation (1–5), exposure time (10–30 seconds), and laser power (0.5–100%; most 5–10%).

#### 2.3.7: Ultra-violet-visible (UV-vis) spectroscopy

Is, to our knowledge, being used for the first time to investigate patinas on historic alloys. While it is a common technique used to identify unknown materials dispersed in solution, the applications of this technique to the analysis of solids refers to pellets obtained with compressed powders. Analyses were carried out on a Cary 5000 UV-vis-NIR spectrophotometer (Varian), available at Laboratoire de Réactivité de Surface, at l’Université Paris-Sorbone, France. The alloys were measured in both specular and diffuse reflection. This allowed a comparison between the two modes, which gave consistent results. The measurements were carried out using a DRA2500 integrating sphere and excluding the specular reflection component from analysis within the sphere. Pristine alloys, and cuprite (cuprous oxide, Cu_2_O) and tenorite (cupric oxide, CuO) powders were measured as reference spectra for the technique.

## 3 – Results

### 3.1: Experimental patinas

#### 3.1.1: Appearance

**Fig 2:**
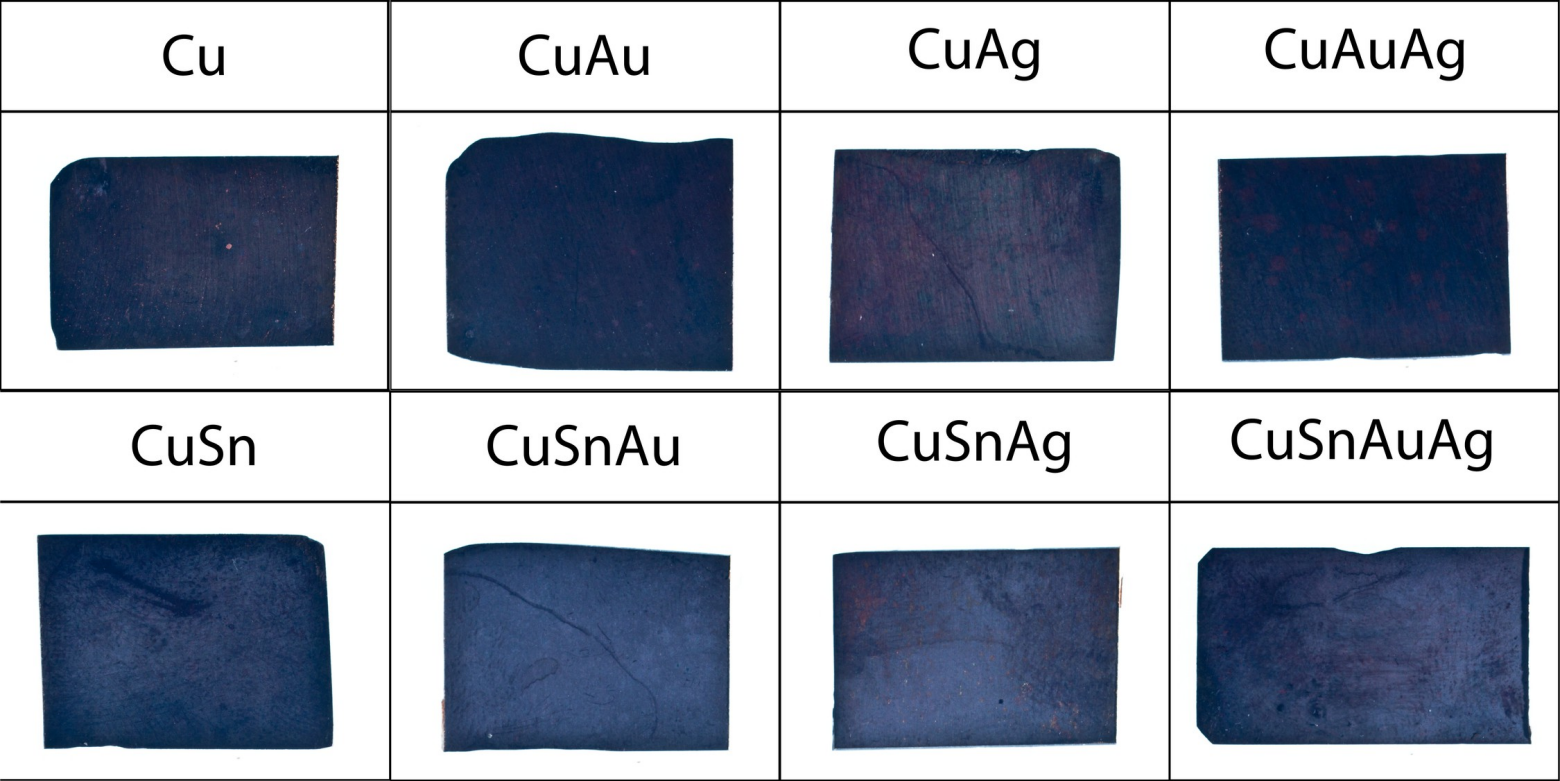
Visual appearance of the patinated alloys. Each rectangle is 2.6 x 1.8 cm.

A dark patina was achieved on all eight alloys (Fig 2). Gold-containing alloys produced darker patinas than alloys without gold, and alloys containing tin resulted in a matte patina, with a powdery appearance and whitish-looking, not corresponding to the dark historical patinas.

**Fig 3:**
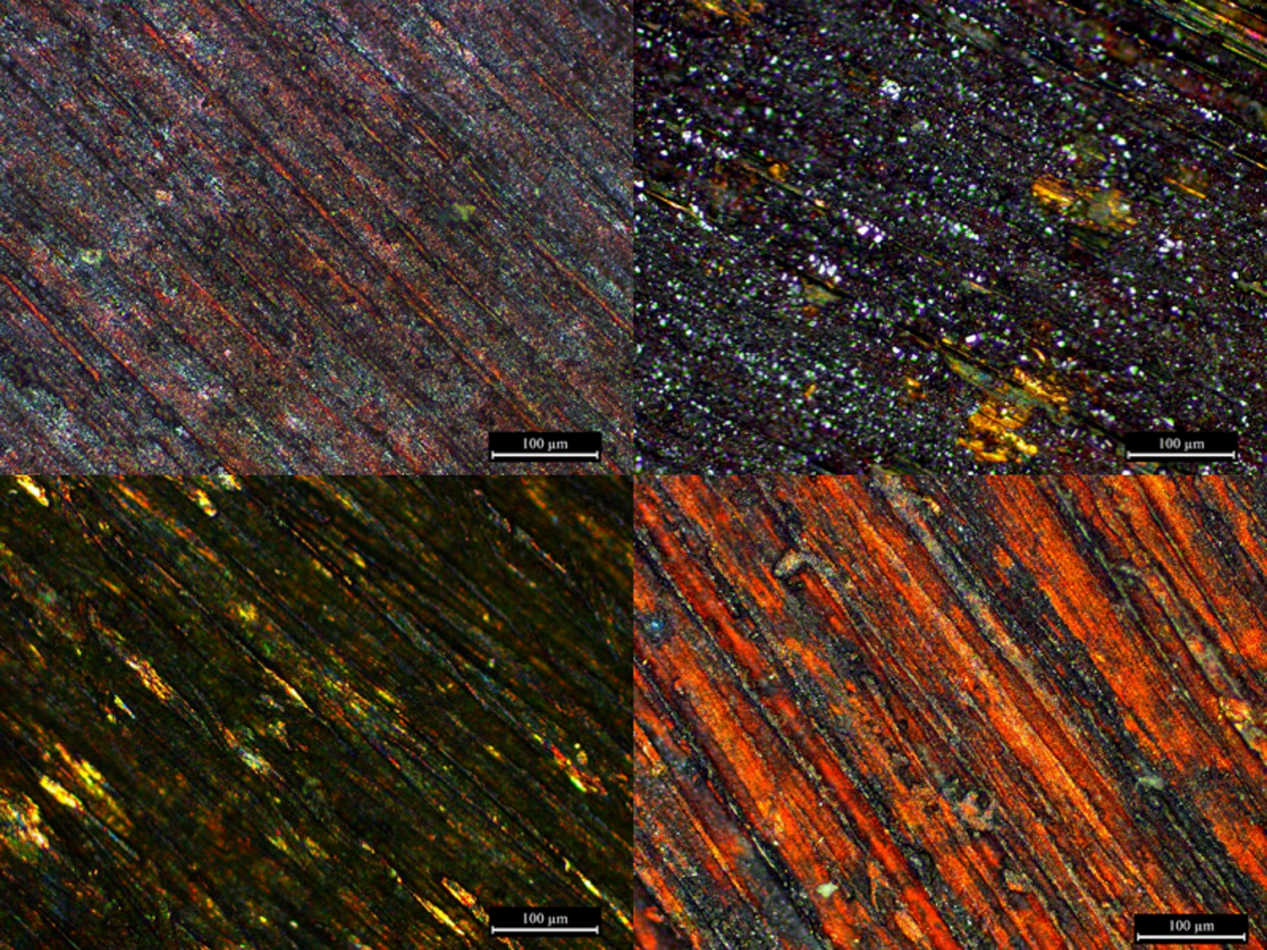
OM pictures showing patinas developed on the alloys without tin (top row) and with tin (bottom row) through chemical patination. Pictures at 50X (each rectangle is 1.2 x 0.9 mm), non-polarised light.

Under the OM (Fig 3), alloys containing silver produced a thin and less uniform patina characterised by patches of red and green, which were darker if the alloy also contained gold (i.e. CuAuAg). Additionally, grinding lines were less visible on alloys containing tin, and were not visible on alloys containing both tin and precious metals; this suggests that a thicker patina developed when tin was present. Patinas with gold appeared bluer and darker than those with silver.

#### 3.1.2: Colorimetric measurements

**Fig 4:**
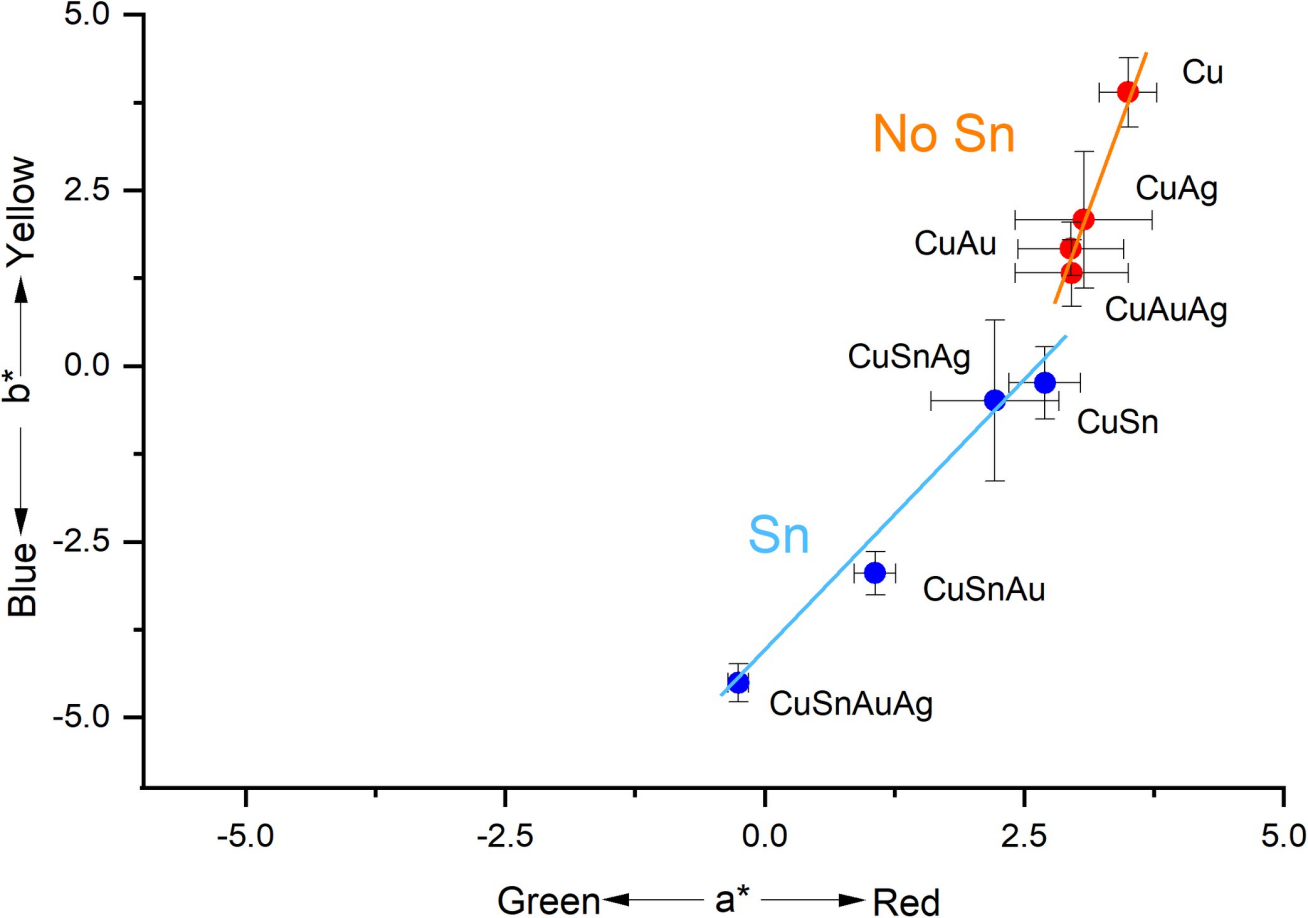
The a*/b* ratio of the patinas made with chemical patination. Each data point represents the mean of five measurements and the error bars represent the standard deviation. Full results are presented in S4 Table in S1 File.

Colorimetric data from alloys without tin where more dispersed on the blue-yellow colorimetric chromaticity coordinate (b*) than on the green-red coordinate (a*), meaning that the redness of a patina changed minimally with composition relative to its blueness (Fig 4). Moreover, the relative standard deviations of the data, calculated on five analyses performed on each token, were higher on the a* values, indicating more variability in this dimension (S4 Table in S1 File). As indicated by the standard deviation, the surfaces of the silver-containing alloys exhibited relatively higher variability in chromaticity coordinates compared to those of gold-containing alloys, irrespective of the presence or absence of tine. Subsequent *pXRF and SEM-EDS* analyses (below) suggest that these differences may be a result of different coverages of the patina on the alloy surfaces.

The alloys tested appear to split colorimetrically into two groups: those containing tin have low b* values, while those without tin have high b* values (Fig 4). Alloys within both groups follow a similar trend from high to low a* and b* values: alloys without noble metals (i.e., Cu and CuSn) > alloys with silver > alloys with gold > alloys with both silver and gold. The presence of tin produces bluer patinas, but there is also a change in the a* value (lower slope; Fig 4). This indicates that the addition of precious metals primarily increases the blueness of patinas, while the presence of tin also increases green shades. Moreover, this analysis demonstrates the role of gold, and to a lesser extent silver, in modifying the colour towards bluer shades.

Although alloys without tin produce patinas with higher b* values than those with tin, L (lightness) values show an opposite trend, with patinas on tin-bearing alloys having higher L values and therefore being less black than alloys without tin (S5 Fig in S1 File). This is consistent with the appearance to the naked eye (Fig 1), where the powdery, matte appearance of the tin-bearing patinas make the resulting surfaces appear lighter. The ∆E values, that quantify the difference between the colours in the CIE L*a*b* colour space, confirm the dissimilarity between alloys without and with tin (S4 Table in S1 File).

#### 3.1.3: Composition and microstructure

The bulk chemical compositions of patinas were assessed with pXRF and SEM-EDS. The pXRF measurement provided a penetration depth estimated to have been in excess of 20 µm and as a result, significant variation in patina composition was difficult to observe owing to large contributions from the underlying bulk alloy. XRF data are provided in S6 Table in S1 File.

**Fig 5:**
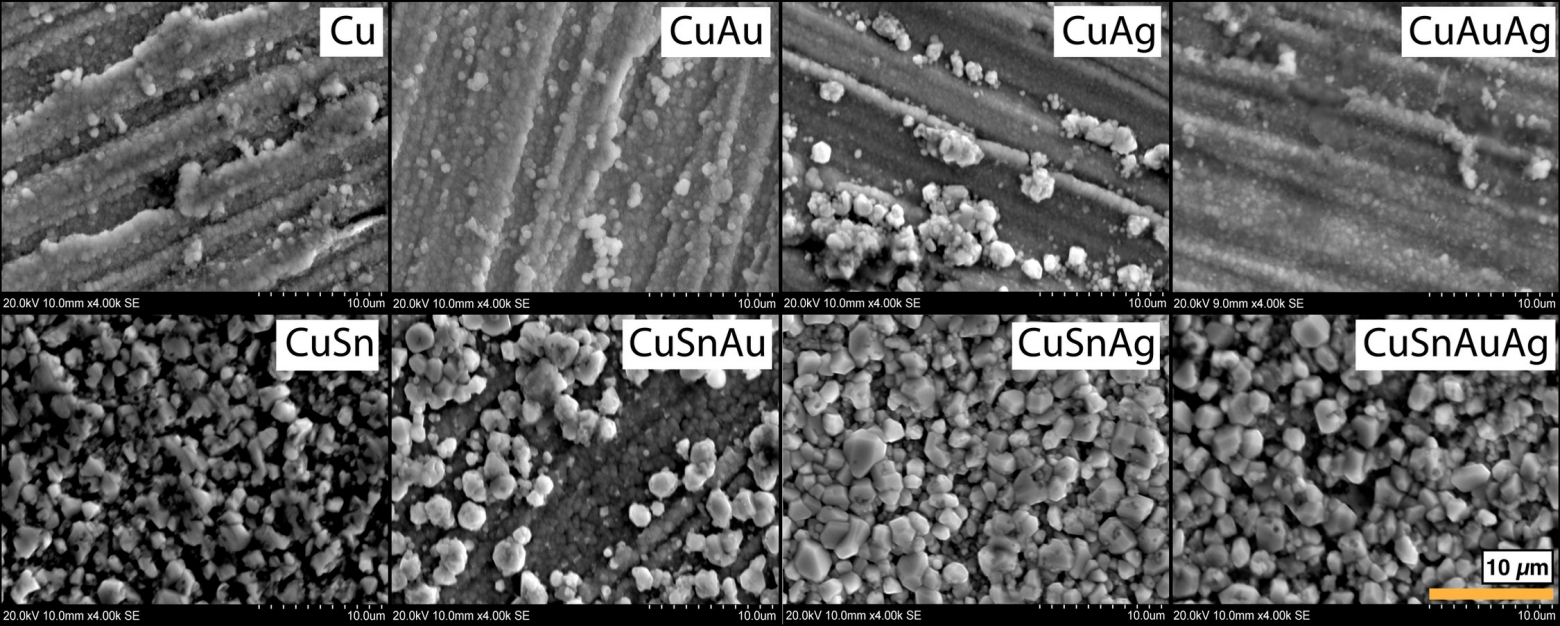
SEM images of the surfaces of the alloys after patination at 4000x (area: 31.5 x 20.0 µm), secondary electron. First row: alloys without tin; second row: alloys with tin. The image shows a substantial difference between the microstructure of the patina on alloys without tin, fine and smoot, and the patinas on alloys with tin, with larger particles.

SEM-EDS samples a more superficial volume (estimated penetration depth less than 10 µm), and therefore proved more useful than pXRF for the close observation of patina composition. The amount of oxygen in the studied alloy flat surface of the unprepared sample was found to increase from 1–2 wt.% before to 30 wt.% after patination, indicating significant oxidation of the alloys; correspondingly, all other elements (Cu, Sn, Au, Ag) in the alloys decreased (Table 1 and S7 Fig in S1 File). SEM-EDS analysis and images suggest that the tin-bearing alloys developed a thicker patina of precipitated cuprite. No gold or silver was detected in the tin-bearing patinas (LoD for gold and silver was around 0.5 wt.%) probably because of the shielding effect of the thicker coating (Table 1 and Fig 5). In contrast, in alloys without tin, it was still possible to detect gold and silver, reflecting the poorer patina coverage on such alloys.

**Table 1:** Surface compositions of the alloys before (columns on left) and after (columns on right) patination. EDS analysis was carried out at 100X magnification (area of analysis: 1.3 x 0.8 mm). Each value is the mean of 3 measurements. Highlighted rows indicate the most common alloy compositions described in historical sources. The presence of oxygen before patination is likely due to the tarnish of the patinas in air occurred from the moment of the creation of the alloy to the day of the analysis.

	Before patination (wt.%)					After patination (wt.%)				
Alloys	O	Cu	Sn	Au	Ag	O	Cu	Sn	Au	Ag
**Cu**_**100**_	1.16	98.84	<LOD	<LOD	<LOD	32.44	67.56	<LOD	<LOD	<LOD
**Cu**_**95**_ **Sn**_**5**_	2.42	92.75	4.83	<LOD	<LOD	27.94	71.68	0.38	<LOD	<LOD
**Cu**_**97**_ **Au**_**3**_	1.82	95.45	<LOD	2.73	<LOD	32.23	67.23	<LOD	0.54	<LOD
**Cu**_**97**_ **Ag**_**3**_	2.38	94.94	<LOD	<LOD	2.68	29.98	68.81	<LOD	<LOD	1.21
**Cu**_**92**_ **Sn**_**5**_ **Au**_**3**_	1.03	91.68	4.49	2.81	<LOD	24.65	74.83	0.52	<LOD	<LOD
**Cu**_**92**_ **Sn**_**5**_ **Ag**_**3**_	1.84	91.17	4.41	<LOD	2.59	24.31	74.58	0.74	<LOD	0.37
**Cu**_**94**_ **Au**_**3**_ **Ag**_**3**_	2.34	92.62	<LOD	2.46	2.58	31.63	66.61	<LOD	0.76	1.01
**Cu**_**89**_ **Sn**_**5**_ **Au**_**3**_ **Ag**_**3**_	1.29	88.52	4.91	2.53	2.74	26.02	73.62	0.36	<LOD	<LOD

Patina coverage of the alloy substrate was assessed using SEM imaging (Fig 5). In alloys without tin, the patina was composed of a fine, compact, irregular morphology adhered to the alloy substrate. The presence of tin in the alloys affected the patina microstructure, resulting in the formation of large (1 to 4 µm) particles of equant appearance on the surface, overlying a uniform oxide layer; this phenomenon was also noted in the CuAg alloy. However, it was not observed in the preliminary experiments conducted with the same solution but with shorter patination times (30 minutes instead of 90 minutes; [17]). It appears, therefore, that the longer patination time allowed the nucleation and growth of large cuprite particles on the surface of the tin-bearing alloys.

Relative to silver-bearing alloys, gold-bearing alloys developed patinas with higher surface coverage of the metal surface, resulting in a more uniform colour.

**Fig 6:**
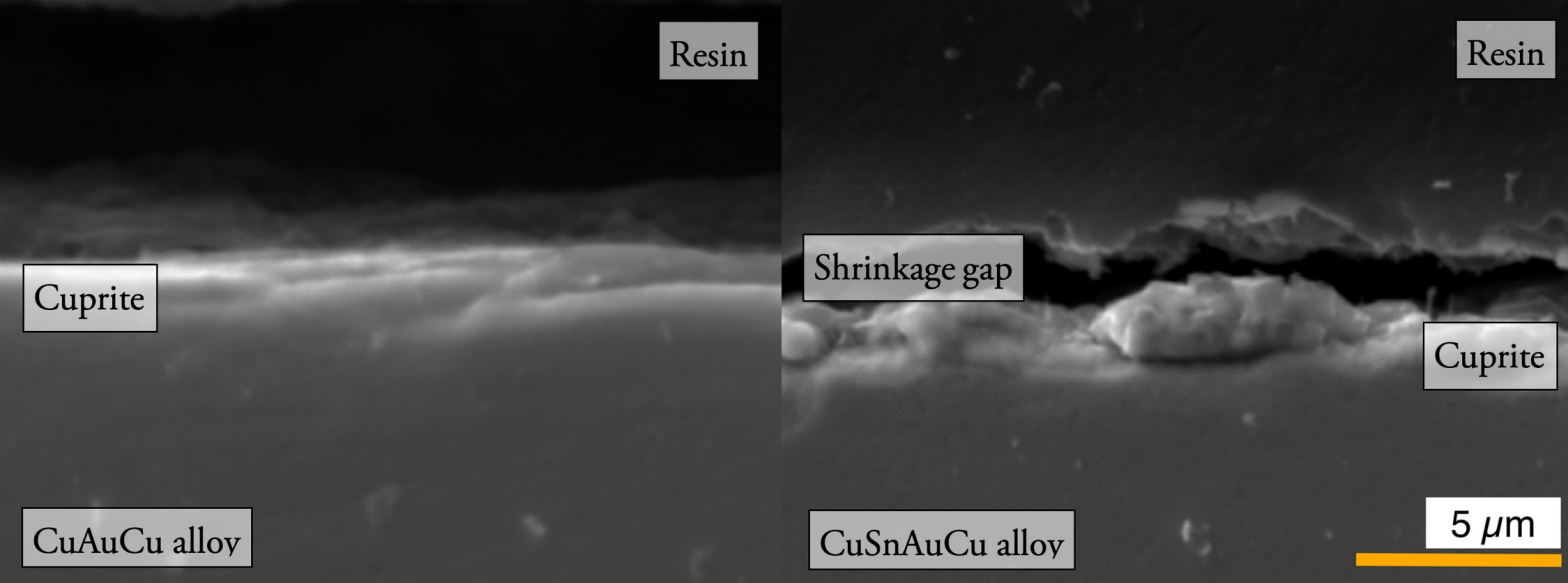
SEM images of the cross section of the alloys after patination at 4000X (area: 31.5 x 20.0 μm), secondary electron. CuAuAg (L), CuSnAuAg (R). On the CuAuAg alloy (L) there is the presence of a thin layer of cuprite only continuous to the alloy. On the CuSnAuAg alloy (R) there is the presence of a further layer of cuprite grains unevenly distributed on the surface of the continuous layer of cuprite alloy.

Cross-sectional analysis (Fig 6) showed compact and uniform patinas on alloys without tin. In accordance with the surface observations, the tin-bearing alloys present a further layer primarily formed of large (~2 µm diameter) cuprite grains. The coverage of this layer is uneven with limited adhesion, leaving areas of exposed substrate. Hence the uniformity of the layer was improved by the presence of precious metals.

The shrinkage of the resin used to prepare polished blocks for the cross-sectional investigation resulted in the detachment of the patina from the surface of the alloy, making it difficult to precisely measure the patina thickness. However, it was still possible to determine the average patina thicknesses within a range. The average thickness measured was between 1 and 2 μm for copper alloys containing precious metals without tin (Cu_97_Au_3_, Cu_97_Ag_3_, Cu_94_Au_3_Ag_3_) and above 2 for the remaining alloys. Excluding pure copper, the patina thickness of the alloys without tin was more homogeneous within the analysed sample. SEM-EDS maps (S8 Fig in S1 File) did not reveal the presence of gold in the patina layer on any alloy.

#### 3.1.4: Phase analysis

The analysis of the phases composing the patinas was completed with XRD, Raman spectroscopy, and UV-vis spectroscopy, which gave consistent results. XRD analysis revealed the presence of both cuprite (Cu_2_O) and tenorite (CuO) in the patina on pure copper, the prevalence of cuprite on tin-bearing alloys, and of tenorite on alloys without tin. These XRD results differ from the results obtained in the preliminary study of Benzonelli *et al*. [17], where the diffraction patterns were less intense, making interpretation difficult. However, as the present patination experiments were significantly longer than the earlier ones (90 versus 30 mins) and as, in the preliminary experiments, the alloys without tin appeared to contain cuprite but not tenorite, it seems likely that the longer patination resulted in further oxidisation of cuprite into tenorite.

**Fig 7:**
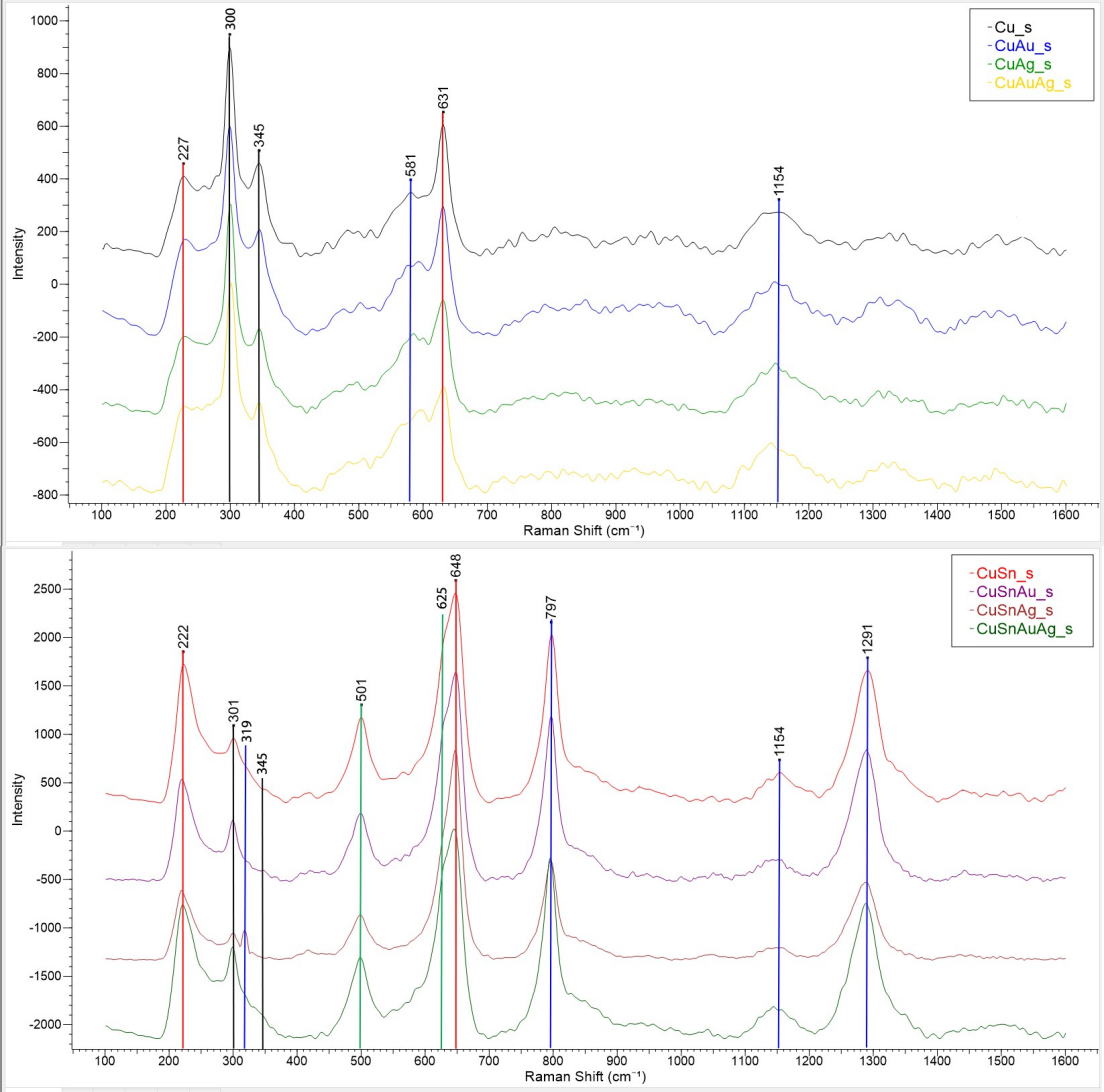
Raman spectra of the patinas obtained by chemical patination on alloys without tin (top) and with tin (bottom). The phases identified are stoichiometric (red lines) and defective (green lines) cuprite, tenorite (black lines) and unidentified peaks (blue lines). Raman signatures from [21, 22].

The Raman spectra from patinas on bronze showed a small presence of tenorite on the patina surface, not detected by XRD (Fig 7). A few peaks remain unidentified.

### 3.2: Museum artefacts

To the naked eye, the patinas of *shakudō* alloys of the three Japanese *tsuba* from the British Museum collections are characterised by a blue-black colour with metallic lustre. No polishing scratches were visible on the surfaces, suggesting that they were polished to a mirror-like finish prior to patination. This was visually assessed to a degree of finish below 4000 grit (5 µm). Lighter and darker patches were observed, likely due to a difference in the patina thicknesses. This variable thickness could have been caused during the patina formation or acquired due to wear. The objects showed only slight signs of wear, however.

The pXRF analysis (full results in S9 Table in S1 File) revealed that five black areas were made of *shakudō*, as they contained precious metals and were artificially patinated (Table 2). These areas make up the bulk of *tsuba* BM-TS.244, the ant of *tsuba* BM1952,0211.31, and the leaf of *tsuba* BM1958,0730.56.d (Table 2). While the first one is made entirely of *shakudō*, the dark base alloys of the last two *tsuba* were made of patinated copper (*tsuba* BM1952,0211.31) and patinated *shibuichi*, a Cu-Ag alloy (Table 2). The gold content in the *shakudō* of these three objects varied from 1.1 to 9.7 wt.%, with silver, arsenic, lead, and iron present as minor elements. It is important to highlight the ubiquitous presence of silver in *shakudo* as well as the anticipated absence of tin.

**Table 2:** Composition of different points of analysis performed on the Japanese artefacts. pXRF analysis carried out using a 5 mm diameter spot, with one measurement in each case. Elements <0.1 wt.%: Ni, Sn, Sb. The full results are reported in S9 Table in S1 File. Highlighted rows indicate the shakudo alloys. Note that in the analysis of small inlays the spot size of the analysis (around 5mm) can include the copper substrate.

Object	Analytical Spot	Description	Code Colorim.	Cu	Au	Ag	As	Pb	Fe
Tsuba–BM-TS.244	1	Bulk	a	97.95	1.08	0.45	0.28	0.17	0.03
	2	Smooth bulk next to blade	b	97.88	1.07	0.46	0.26	0.28	<LoD
	3	Copper		99.45	<LoD	<LoD	0.08	0.36	0.07
	4	Gilded animal–bottom left		4.81	93.87	1.33	<LoD	<LoD	<LoD
	5	Blue flower–bottom left		7.01	1.21	90.11	<LoD	0.35	0.26
	6	Blue flower–top left		22.30	5.41	71.80	<LoD	0.25	0.14
	7	Animal–top left		88.49	9.74	1.10	0.23	0.20	0.03
	8	Leaf–top right		93.03	4.67	1.49	0.26	0.24	0.07
	9	Grape–right		31.32	1.16	67.03	0.00	0.26	0.10
Tsuba BM1952,0211.31	10	Bulk	c	99.18	<LoD	<LoD	0.36	0.40	<LoD
	11	Smooth bulk	d	99.30	<LoD	<LoD	0.30	0 .33	0.01
	12	Copper		99.45	<LoD	<LoD	0.06	0.39	0.04
	13	Head big insect–bottom left		69.80	0.17	29.41	<LoD	0.31	0.00
	14	Body big insect–bottom left		70.44	0.19	28.59	<LoD	0.31	0.12
	15	Gilding		39.06	59.28	1.34	0.15	0.16	<LoD
	16	Ant–top	e	94.14	3.87	1.19	0.26	0.22	0.22
	17	Ant–bottom		96.27	2.17	0.59	0.35	0.39	0.21
Tsuba BM1958,0730.56.d	18	Bulk	f	80.37	<LoD	19.08	0.02	0.27	<LoD
	19	Below blade hole	g	80.02	<LoD	19.44	0.02	0.26	0.03
	20	Red Cu flower–bottom		93.68	1.00	4.80	<LoD	0.36	0.09
	21	Black leaf–right	h	90.03	2.04	7.18	0.29	0.21	0.13
	22	Gold leaf–top right		46.30	35.43	17.90	<LoD	0.16	<LoD

**Fig 8:**
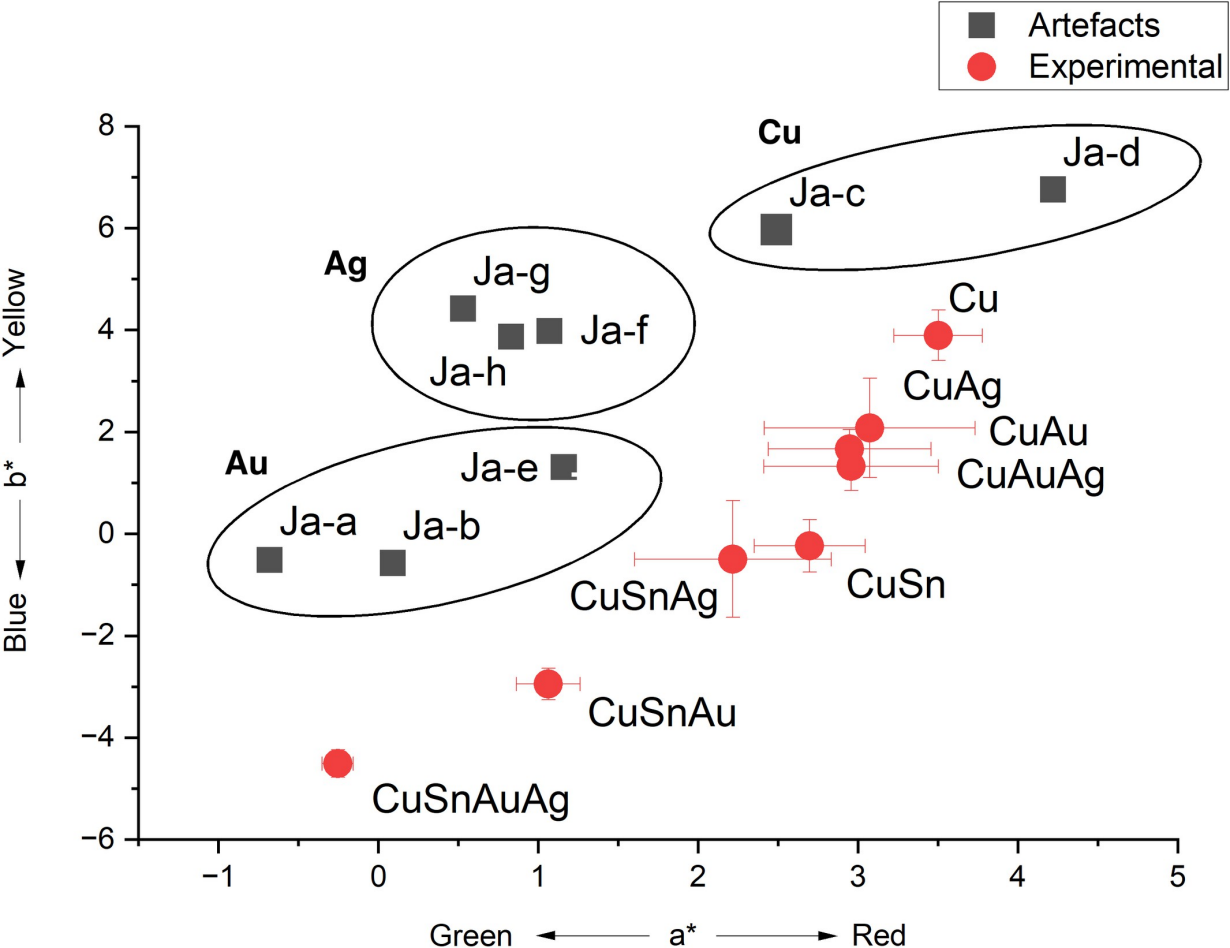
The a* and b* values of the analysed patinas on the Japanese artefacts (black, square symbols) and of the experimental patinas (red, dot symbols). The ellipses represent groups of similar composition. Error bars indicate standard deviation of 5 measurements.

The results of the colour measurements of the dark areas are shown in Fig 8. The *shakudō* alloys were found to have a lower b* value than patinated copper and the patinated copper-silver (*shibuichi*) alloy. *Shakudo* spot Ja-h, which lies in the CuAg ellipse In Fig 8, is characterised by a high amount of silver (7.18 wt.%), together with 2.04 wt.% of gold (see Table 2), probably because of contamination from an adjacent area of Cu-Ag alloy, and consequently has a higher b* value. The presence of silver in the alloy, therefore, was associated with a shifting of the colours of the gold-bearing patinas towards yellower shades.

Compared to the experimental patinas, the colours of the patinas of these historical artefacts were found to have lower a* values, or more greenish colours. Similarly, their a* values were more widely dispersed, though they displayed a similar trend to that seen in the experimental patinas (Fig 8). The spot Ja-e had a similar blue value to that of the experimental sample CuAu, and similar gold content (3.87 wt.% in the historical alloy, 3 wt.% in the experimental patina). Spots Ja-a and Ja-b were bluer and characterised by an alloy with lower gold content (Table 2). As in the experimental materials, CuAg alloys in the artefacts were found to have higher b* values than CuAu alloys.

## 4 – Discussion

### 4.1: Effect of tin

Scientific and historical sources reporting the compositions of the alloys used to produce *shakudō* and *shibuichi* (Cu-Au and Cu-Ag alloys, respectively) do not report tin content (e.g. [3–5, 8, 11]) (although some experiments aimed at reproducing Japanese patinas have been performed on tin-bearing alloys, e.g. [23]). The present experimental work shows a substantial difference between patinas achieved on alloys without and with tin, which would explain the selection of the former.

**Fig 9:**
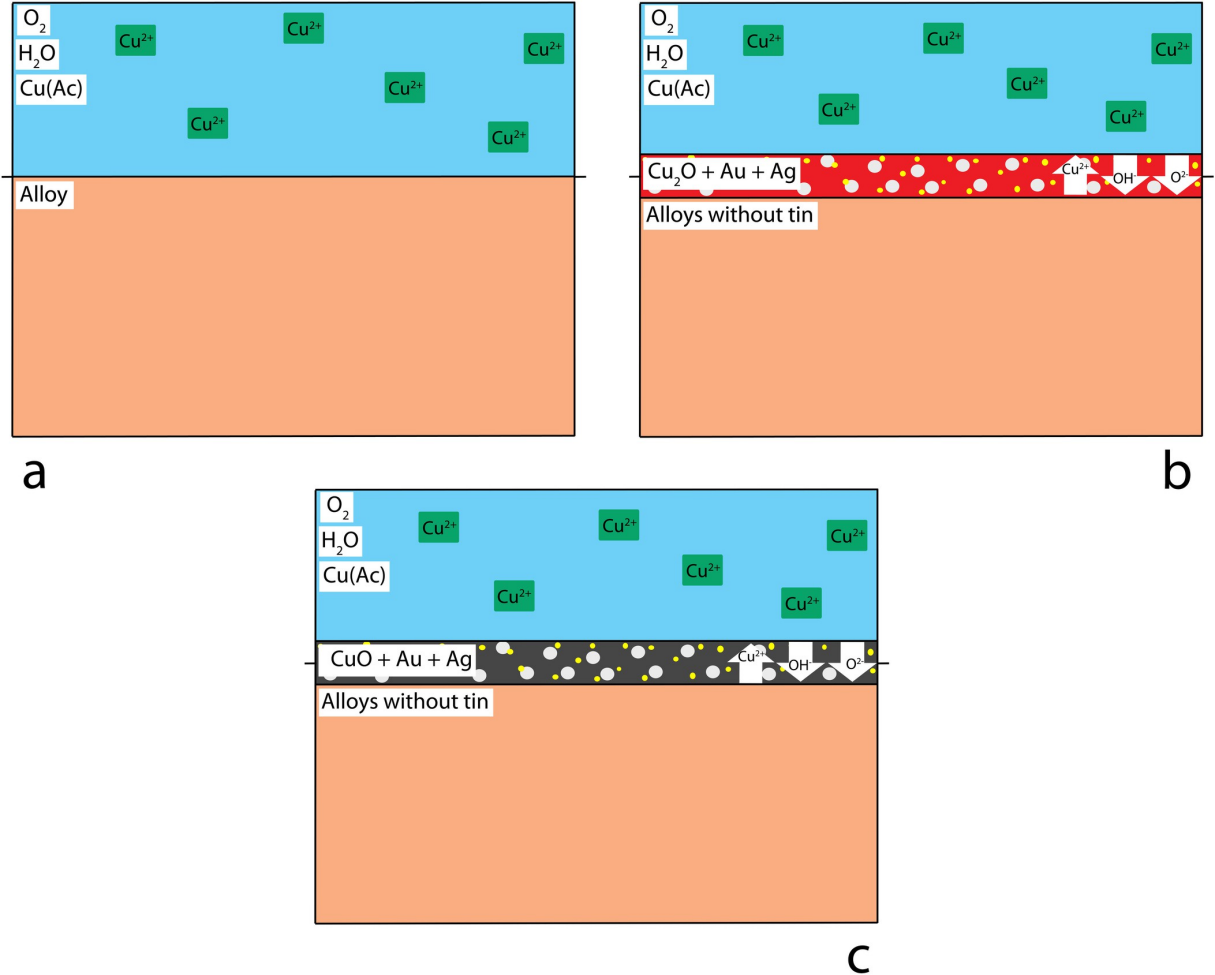
Chemical patination mechanism for samples not containing tin. The presence of Au and Ag particles is proposed in the cuprite patina immediately over the solid-solution alloy substrate, not visible in the experiments performed. (a) before patination, (b) short patination, c) long patination.

In alloys without tin, the surface of the alloy (Fig 9A) oxidises, forming cuprite (Fig 9B). A cuprite patina is compact and passivating; with time, a suitable thickness is achieved to limit additional ion transport. However, while a cuprite patina was achieved in the preliminary tests performed for 30 minutes [17] a longer patination time (90 minutes, this paper) caused cuprite to be converted into tenorite (Fig 9C). This does not reflect the characteristics of the historical patinas which are all reported in literature to contain cuprite only, suggesting the use of short patination times. Longer patination times would also affect the particle size of the patina due to crystal growth or Ostwald ripening, supporting the suggestion that a patina of limited thickness was the result required.

Earlier proposals report that it is likely that gold and silver are present in the form of nanoparticles in the cuprite patina seem possible (Fig 9B), but it was not possible to assess their presence with the use of SEM-EDS. The presence of nanoparticles, their form and quantification should be assessed in further analysis.

The standard electrode potential is higher in gold, followed by silver, copper and tin [24]. As seen in the Pourbaix diagram [25], Cu behaves as a noble metal and is in the passive regime for all pH values at 100°C. Cu stability increases still further for lower temperatures, including the treatment temperature of 80°C [25, p. 479].

**Fig 10:**
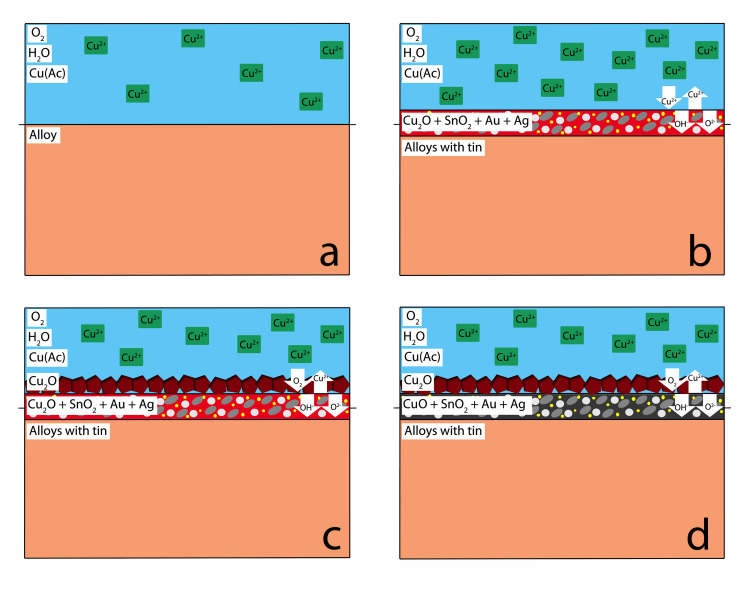
Chemical patination mechanism for samples containing tin. Large tin oxide (SnO_2_), Au, and Ag particles in cuprite form the patina over the solid-solution alloy surface. (a) before patination, (b-c) short patination, d) long patination.

Similar to alloys without tin, in tin-bearing alloys the surface of the alloy (Fig 10A) is oxidised to form a cuprite layer (Fig 10B), shakudo containing oxidised tin, metallic gold, and metallic silver as nano or sub-microscopic particles below the SEM-EDS resolution. Cassiterite (SnO_2_), a mineral usually present in historical corroded bronzes, was not detected using either XRD or Raman spectroscopy. The failure to detect SnO_2_ may be either because it was non-stoichiometric or because it was present at the interface between the metal and the metal oxides, therefore too deep to be detected.

Of the alloying elements, tin is the most reactive. Its addition reduces the standard electrode potential and hence the alloy corrosion resistance. However, since additions of tin are limited to 5 wt.% in our experiments, this effect is expected to be limited [25, p. 479].

Metallic Sn will dissolve at low pH to form SnOH^+^, although these cations will quickly stabilize as SnO_2_, providing passivation protection from additional corrosion attack. The addition of Sn induces significant roughness in the layer formed and broadens the pore-size distribution, while tin-free alloys develop uniform patina layers, suggesting that copper oxides precipitated onto the alloy surface of tin-bearing alloys.

The tin in the alloy also promotes Cu dissolution [26], also due to the strength of the Sn-Cu bond, which is weaker than the Cu-Cu bond (Table 3). These findings are in agreement with Robbiola’s proposed mechanisms of corrosion in tin-bronze alloys [27].

**Table 3:** Bond dissociation energies. Data from [30].

Bond	Energy
Ag-Sn	136
Ag-Ag	163
Ag-Cu	176
Cu-Sn	177
Sn-Sn	195
Cu-Cu	202
Ag-Au	203
Au-Au	221
Au-Cu	232
Au-Sn	244

It is therefore proposed that in tin-bearing alloys the higher rate of release of copper ions results in a more pronounced concentration gradient at the solid-liquid interface, which promotes the growth of larger cuprite polyhedral crystals on the surface of the metal (Fig 10C). Larger oxide particles formed on the metal surface can also be seen in CuAg alloys, probably because the Ag-Cu bond energies are similar those of the Sn-Cu bonds (Table 3). This coarse layer is porous and allows additional oxidation of the inner layer into tenorite (Fig 10D). The coarser patina morphology also makes this patina look ‘powdery’ and whitish (Fig 2), diffracting more of the light on the surface. The newly formed cuprite layer on the surface does not contain gold or silver, as shown in the SEM-EDS results (Table 1).

In sum, while the previous experiments [17] showed solution composition to be the main controlling factor for the resulting patina microstructure, the new tests also demonstrate that alloy composition, particularly the presence or absence of tin, significantly affects the final microstructure and the phases present, and hence the patina properties.

### 4.2: Presence of gold

The influence of gold content on *shakudo* patina colours was previously studied by several authors [e.g. 3, 4, 12, 28], who found that a decrease in gold content corresponded with an increased spectral reflectance of a patinated object in the red region (600–759 nm). *Shakudō* with percentages of gold between 0.25 and 3 wt.% developed a black or brown colour after patination; those with higher gold showed a black colour with purple reflections [4].

Our colorimetric analysis of experimental *shakudo* and *shibuichi* has shown how the presence of gold affects the final patina colour (Fig 4). Adding gold and silver moves the colour of the final patina towards green (lower a* values), and adding gold moves the colour more towards blue (lower b* value) than silver additions alone. The patinas obtained on gold-rich and silver-rich alloys are characterised by different b* values, while their a* values are similar.

From a chemical point of view, in contrast to tin, the addition of silver and gold will shift the standard electrode potential of the alloy to render it less reactive, improving the alloy corrosion resistance. The resulting patinas develop more slowly and are thinner and more compact. Further, the present experiments have shown that added gold renders the patina more homogeneous, uniform, and black. This seems a clear indication that gold was used intentionally to achieve the desired result, and that this use of gold was an intrinsic part of their patina-creation technology, and that ‘sacrificing’ gold in the making of a dark patina was well justified for technical and practical reasons.

### 4.3: Artefacts analysis and comparison with experimental results

The analysis of archaeological artefacts showed that the surface is smooth, without observable crystals or scratches. This supports the idea that the alloys underwent a fine degree of polishing prior to patination, but an analysis of the surface of the artefacts using SEM is required to confirm this finding.

In contrast with recipes reported in scientific literature [e.g. 5, 29], the *shakudo* alloys analysed contain both gold and silver, with the latter in lower percentages compared to gold.

In agreement with the experimental results, the colorimetry analysis on historical artefacts indicates that the colour is affected by the presence of gold and silver (Fig 8). As in the experimental patinas, the patinas obtained on Au-rich and Ag-rich historical alloys are also characterised by different b* values, while their a* values are similar.

The colour measured on the historical artefacts have the same trend of the one measured on experimental patinas, with patinas on alloys containing mainly gold bluer than the patinas on alloys containing mainly silver, which are in turn bluer than the patinas on alloys without precious metals.

### 4.4: The best alloy

For centuries, Japanese craftspeople have had a robust empirical understanding of their materials, and a wide range of potential choices to make a black patinated alloy. However, for their most outstanding pieces, their choices were remarkably consistent: in the case of *shakudo*, they invariably went for copper alloys containing gold but not tin. The observations made in the present experiments, which are fully consistent with those on the objects from the British Museum, provide explanations for the alloy choices. A good patina needs not only to yield the desired colour, but also to have appropriate reflectance, a fine, smooth surface, and to adhere well to the metal substrate. This work has shown that while the presence of tin can produce a potentially acceptable black patina colour, it promotes a more rapid corrosion of the metal, which leads to the growth of large cuprite crystals and a more matt and powdery surface, with poorer adherence. The addition of silver and particularly gold on the other hand reduces the corrosion rate, leading to a slower growth and a thinner, more compact patina, which adheres well to the substrate. Long chemical treatments tend to produce thicker, less adhesive patinas and oxidise the cuprite of the patina to tenorite, which does not adhere well to the surface. Moreover, tenorite does not have the similar blue shade typical of the *shakudo*, and it has not been found on historical artefacts.

In sum, it is evident that the choices made in the selection of the components of the alloys by the Japanese craftspeople are all converging on a reduced corrosion rate that leads to the formation of a thin, adherent blue-black patina on the surface of the artefacts.

## 5 – Conclusions

The comparison between experimental patinas obtained with alloys known to be used from historical sources with alternatives not historically used, and between historical and experimental patinas, has helped our understanding of the technological choices made by Japanese craftspeople in the alloy selection. In particular, compared to the other alloy compositions, the absence of tin and the presence of gold limit the growth of an oxide layer and promote the formation of a thin patina characterised by a smooth appearance without visible grains. Therefore, limiting the thickness of the patinas is a key aspect for the production of the desired colour and appearance of the patinas.

The first colorimetric analysis on historical Japanese artefacts demonstrates the influence of gold, silver and tin in the final patina colour, validating the observations in the experimental replicas.

SEM-EDS analysis on the surface of museum Japanese artefacts should be performed to better assess the presence of the diagnostic features seen in the experimental project, such as microstructure of the surface, and the absence of crystals at the microscopic level.

An outstanding issue remains the presence/absence of noble metal nano particles in the patinas. Our techniques would not have allowed us to observe these, but we note that we have been able to explain many of the characteristics of the patination process without invoking nano particle formation. Further investigation is required to address this problem, and the eventual role of the nanoparticles of precious metals in the patina and colour formation.

## Supporting information

S1 File (ZIP)
