# Supplementary material for: Effects of alloy composition in traditional Japanese shakudo patination
Source: PLoS One. 2023 Nov 17;18(11):e0289728. doi: 10.1371/journal.pone.0289728 (PMC10656006; doi:10.1371/journal.pone.0289728)
Supplement: S1 File — (ZIP) [file pone.0289728.s001.zip › Supporting Information/Supporting Information.docx]

**Supporting Information**

**Table S1:**

| Artefact | Museum n. | Department | Date | Material(s) | Measure | Body/inlay |
| --- | --- | --- | --- | --- | --- | --- |
| Tsuba | TS.244 | Asia | 17th C | Shakudo, silver, gold, copper | L: 7.1 cm | Body |
| Tsuba | 1952,0211.31 | Asia | 18^th^ C | Shakudo, shibuichi, gold, copper | L: 6.9 cm | Inlay |
| Tsuba | 1958,0730.56.d | Asia | 1868-1912 | Shakudo, shibuichi, gold, copper | n/a | Inlay |

Table S1: Details of the artefacts analysed at the British Museum.

**Table S2:**

See Excel file

*Table S2: pXRF analysis of CRMs and LOD.*

**Table S3:**

See Excel file

*Table S3: SEM-EDS analysis of CRMs.*

**Table S4:**

| Alloy | L | SD | a* | SD | b* | SD | Alloy | L | SD | a* | SD | b* | SD | ∆E |
| --- | --- | --- | --- | --- | --- | --- | --- | --- | --- | --- | --- | --- | --- | --- |
| Cu | 25.51 | 0.76 | 3.50 | 0.28 | 3.90 | 0.49 | CuSn | 28.39 | 3.50 | 2.70 | 0.35 | -0.24 | 0.51 | 5.10 |
| CuAu | 24.70 | 0.56 | 2.95 | 0.51 | 1.67 | 0.38 | CuSnAu | 38.92 | 0.18 | 1.06 | 0.20 | -2.95 | 0.31 | 15.07 |
| CuAg | 26.85 | 2.50 | 3.07 | 0.66 | 2.08 | 0.97 | CuSnAg | 35.92 | 2.37 | 2.22 | 0.61 | -0.49 | 1.14 | 9.46 |
| CuAuAg | 25.95 | 0.82 | 2.96 | 0.55 | 1.32 | 0.47 | CuSnAuAg | 26.57 | 1.42 | -0.25 | 0.10 | -4.51 | 0.27 | 6.68 |

Table S4: Surface colour in the L*a*b* colour space of the alloys after the patination. Mean and standard deviation of 5 measurements.

**Figure S5:**


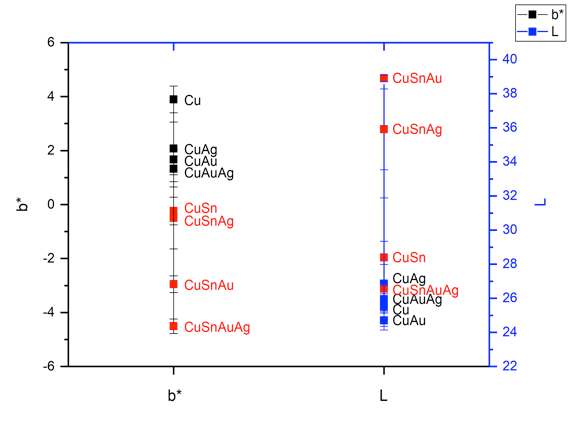


Fig. S5: b* (black axis and data points) and L* (blue axis and data points) plot of the patinas made with chemical patination. Each data point represents the mean of five measurements. Sn-bearing alloys indicated in red.

**Table S6:**

**
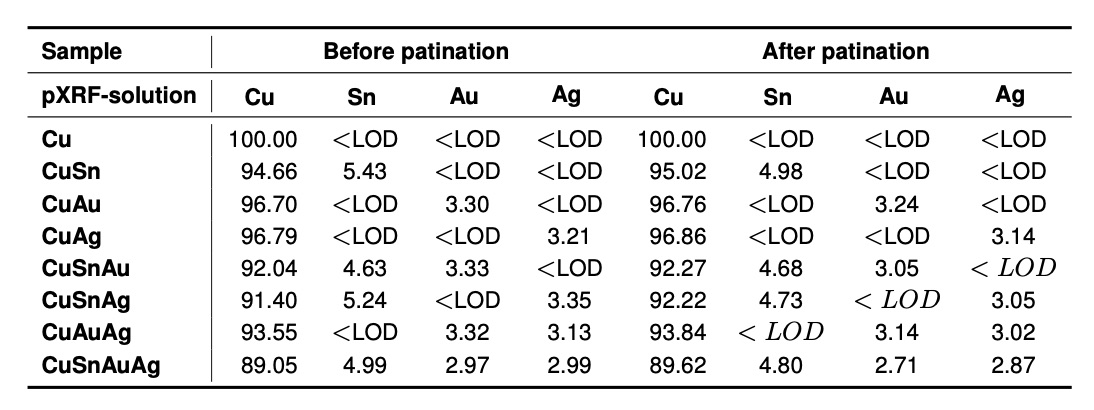
**

Table S6: Surface composition of the alloys before (left-hand side) and after (right-hand side) the patination. pXRF analysis carried out with 3mm diameter spot. Mean of 4 measurements.

**Figure S7:**


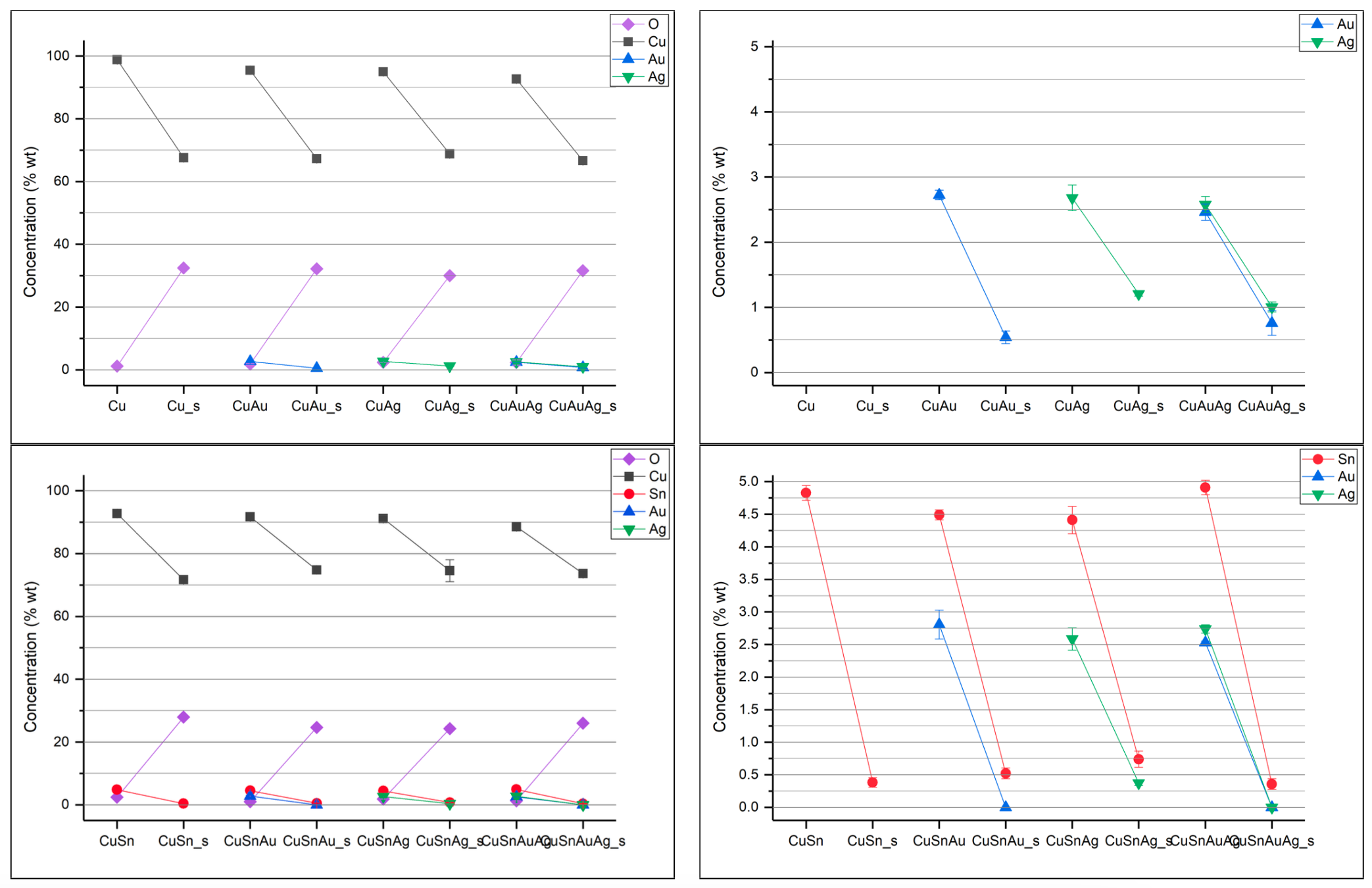


*Fig. S7: Scatter plot of SEM-EDS analyses of tokens before and after patination, showing alloys without tin (top row, left) and alloys with tin (bottom row, left). Details of the graphs appear on the right. Each point represents the mean of three measurements; error bars represent one standard deviation.*

**Figure S8:**

**
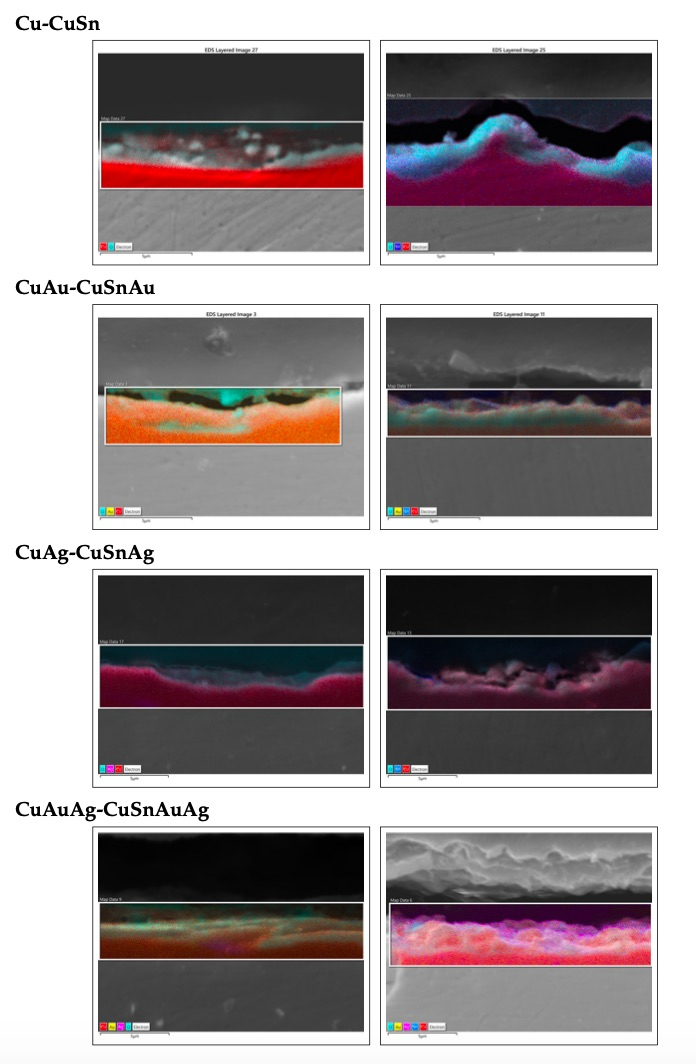
**

*Figure S8: X-ray maps of the samples treated with Japanese patination.*

**Table S9:**


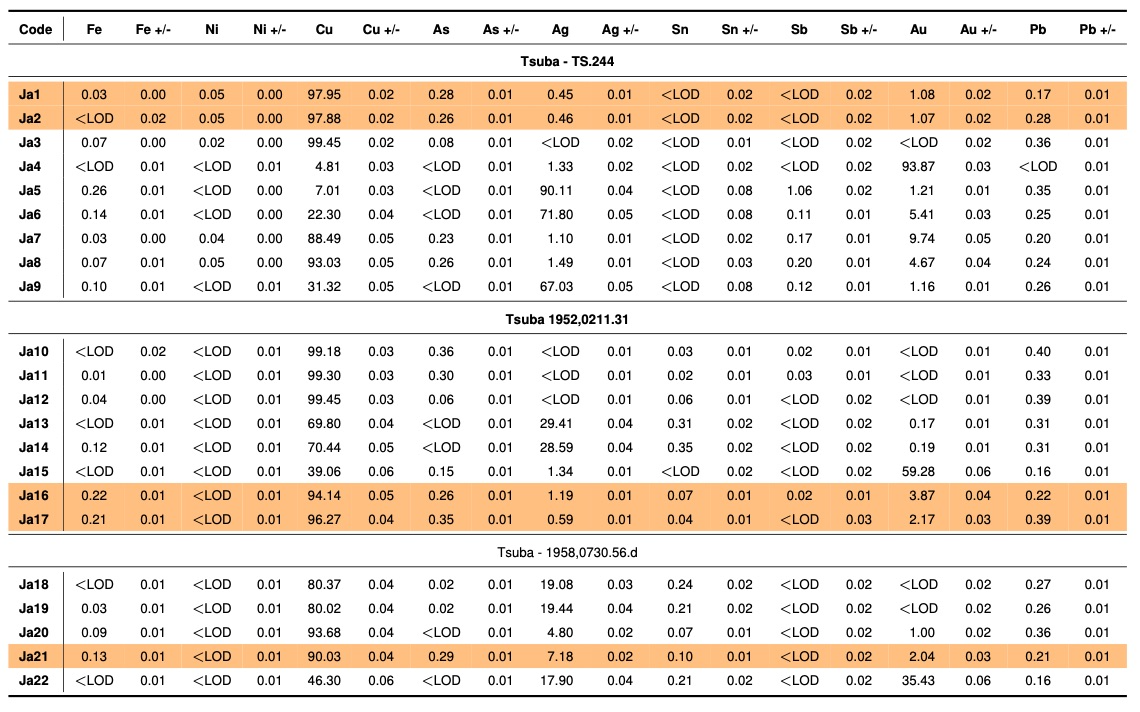


*Table S9: Results of the XRF analysis performed on the Japanese artefacts, carried out with 3 mm diameter spot, one measurement. Elements <0.1%: Ni, Sn, Sb. The rows highlited in orange show the shakudo. It was not possible to perform analysis on the golden areas because they were smaller than the collimated beam of the pXRF.*
